# Establishment and Comparative Analysis of Enzyme-Linked Immunoassay and Time-Resolved Fluoroimmunoassay for the Determination of Trace Quinclorac in Environment

**DOI:** 10.3390/bios12050338

**Published:** 2022-05-14

**Authors:** Xue Liu, Xiuzhai Chen, Xu Zhu, Qing Lin, Xi Pan, Xiaolei Tan, Yongfeng Guo, Jun Qiu, Song Fang

**Affiliations:** 1 Tobacco Research Institute of Chinese Academy of Agricultural Sciences, Qingdao 266101, China; liuxue01@caas.cn (X.L.); guoyongfeng@caas.cn (Y.G.); 2 Shandong Linyi Tobacco Co., Ltd., Linyi 276003, China; yang99s@126.com (X.C.); 18369590005@163.com (X.Z.); whxiaolei123@126.com (X.T.); 3 Nanping Tobacco Company of Fujian Province, Nanping 353000, China; linqing7806@163.com; 4 Sanming Tobacco Company of Fujian Province, Sanming 365000, China; pannix7@163.com

**Keywords:** quinclorac, enzyme-linked immunoassay, time-resolved fluoroimmunoassay, residue analysis

## Abstract

As a common herbicide in farmland, there has been wide concern over quinclorac residue because of its potential risks to the environment and human health. For the detection and monitoring of quinclorac residue in the environment, enzyme-linked immunoassay (ELISA) and time-resolved fluoroimmunoassay (TRFIA) were established. The half-maximal inhibition concentrations (IC_50_) of ELISA and TRFIA were 0.169 mg/L and 0.087 mg/L with a linear range (IC_20_–IC_80_) of 0.020–1.389 mg/L and 0.004–1.861 mg/L, respectively. Compared with ELISA, the limit of detection (LOD, IC_20_) and IC_50_ of TRFIA improved approximately 5-fold and 2-fold. The cross-reaction rates for the quinclorac analogs were less than 2%. The average recoveries of quinclorac in river water, paddy water, paddy soil, and brown rice samples were 77.3–106.1%, with RSDs of 1.7–12.5%. More importantly, the results of the two methods were consistent with that of the referenced method of UPLC-MS/MS (R^2^ > 0.98). ELISA and TRFIA both showed good detection performance and could meet the requirements of the quantitative determination of quinclorac. Therefore, the proposed ELISA and TRFIA could be applied to the rapid and sensitive detection and monitoring of quinclorac residue in the environment.

## 1. Introduction

Herbicide application is the most effective way to control weeds and ensure food production [1,2]. However, herbicide residues in the field might cause serious environmental pollution, which affects the quality and safety of agricultural products and, thus, harms human health [3,4,5]. Quinclorac is a disubstituted quinoline carboxylic acid hormone-type herbicide, which is used to selectively control weeds, such as barnyard grass in paddy fields and sorghum fields [6]. In recent years, there has been wide concern over quinclorac residues because of its high dosage in usage, long half-life, and high risk to succeeding crops and the environment. First of all, quinclorac has been at the forefront of herbicide applications in paddy fields worldwide; however, the long-term continuous use of quinclorac leads to weed resistance [7,8]. Secondly, the long half-life (long retention time) of quinclorac increases the risk of environmental safety [9,10]. Finally, quinclorac residue is prone to cause phytotoxicity to certain succeeding crops such as tobacco, potato, carrot, and spinach, which are extremely sensitive to quinclorac [11,12]. Therefore, there is an urgent need to apply appropriate detection methods to monitor quinclorac residue in the environment.

Currently, high-performance liquid chromatography (HPLC) is the most commonly used method for the determination of quinclorac. HPLC coupled with ultraviolet (UV) or mass spectrometry (MS) to determine quinclorac residues in different matrices have been reported [13,14]. Although these instrumental methods are sensitive and accurate, they suffer from some drawbacks such as consuming a large amount of organic solvent, time-consuming procedures, and high maintenance and operation costs. In contrast to chromatographic analysis, a simple and rapid determination of quinclorac in rice samples has been reported thanks to the advantages of QuEChERS (quick, easy, cheap, effective, rugged, and safe) extraction and fluorescent analysis [15]. However, the detection limit appeared to fail to meet the requirements for the determination of trace quinclorac in the environment. Therefore, it was urgent to establish a simple, rapid, and sensitive method for the determination of quinclorac.

Compared with the instrumental methods for detecting environmental pollutants, antibody-based immunochemical technology has shown great advantages of high efficiency, rapidity, reliability, and low costs [16,17,18]. In recent years, the enzyme-linked immunoassay (ELISA) has been established for the rapid and high throughput screening of a large number of samples [19,20]. Based on the use of lanthanide chelates as fluorescence labels, the time-resolved fluoroimmunoassay (TRFIA) is one of the most sensitive fluorescence immunoassays. Lanthanide ions (such as Eu^3+^) could provide the benefit of temporal resolution of a signal from the background, dissociating the labels by a pH shift into new highly fluorescent chelates, a substantial Stokes’ shift, and narrow emission peaks; thus, higher sensitivity and lower interferences for the detection methods might be developed [21,22,23,24]. To the best of our knowledge, only two reports that studied the synthesis and identification of the artificial antigen of quinclorac are available [25,26]. To date, no report about the application of ELISA and TRFIA in the analysis of quinclorac in the environment has been found.

In this study, an artificial antigen of quinclorac by conjugating the hapten of quinclorac with a carrier protein was designed and synthesized. Monoclonal antibodies (MAb) against quinclorac were obtained from immunized mice. ELISA and TRFIA of quinclorac were established by optimizing the experimental parameters, and the results were compared with that of ultra-performance liquid chromatography tandem mass spectrometry (UPLC-MS/MS). The established ELISA and TRFIA were applied to the rapid, sensitive, and specific detection of quinclorac in environmental samples.

## 2. Materials and Methods

### 2.1. Materials

Quinclorac and subsequent analogs (7-chloro-8-methylquinoline, 2-quinoline carboxylic acid, quinmerac) were purchased from Shanghai Yuanye Bio-Technology Co. Ltd. (Shanghai, China). *N′*-[*p*-Isothiocyanato-benzyl]-diethylene-triamine-*N^1^*,*N^2^*,*N^3^*,*N^4^*- tetraacetate-Eu^3+^ (DTTA-Eu^3+^) was purchased from Tianjin Radio-Medical Institute (Tianjin, China). Bovine serum albumin (BSA), ovalbumin (OVA), affinity-purified goat antimouse IgG (GAM-IgG), horseradish peroxidase-labeled goat antimouse IgG (HRP-GAR-IgG), 3,3′,5,5′-tetramethylbenzidine (TMB) solution (0.4 mmol/L, in citrate buffer, pH 5.0) were purchased from Sigma Chemical Co. (Shanghai, China). Enhancement solution was provided by Jiangsu Nuclear-Medicine Institute (Wuxi, China). Other reagents (analytical grade) were purchased from Keyuan Biotech (Qingdao, China).

Buffer solutions used in this study: Phosphate buffered saline (PBS, pH 7.4, 0.01 mol/L), carbonate buffered saline (CBS, pH 9.6, 0.05 mol/L), PBS containing 0.05% Tween-20 (PBST), and Tris-HCl buffer (TBS, pH 7.8, 0.05 mol/L) containing NaCl (0.01%).

### 2.2. Instruments

Nuclear magnetic resonance (NMR) was detected by a DRX500 spectrometer (Bruker, Karlsruhe, Germany). UV absorbance was detected by using a NanoDrop-1000 spectrophotometer (Thermo, Wilmington, DE, USA). The fluorescence was determined by an M200 microtiter plate reader (Tecan, Männedorf, Switzerland). The proposed ELISA and TRFIA were confirmed by an ultra-performance liquid chromatography tandem mass spectrometer (UPLC-MS/MS, 4500 Orbitrap, AB SCIEX, Foster City, MA, USA).

### 2.3. Hapten Synthesis and Artificial Antigen Preparation

**Figure 1:**
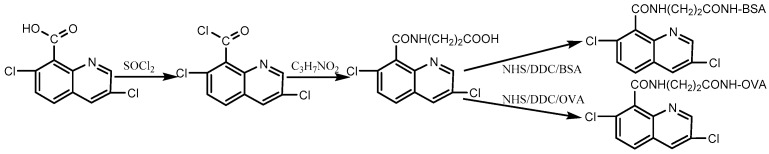
The preparation route of the quinclorac hapten and artificial antigen.

The routes of the hapten and artificial antigen preparation are shown in Figure 1. Hapten was synthesized as previously described with some modifications [25]. Briefly, 3 mmol of quinclorac was added to the three-necked flask, followed by 15 mmol of thionyl chloride. The mixture was heated and refluxed at 100 °C for 2 h and then concentrated at 50 °C under reduced pressure to obtain the intermediate. The intermediate was dissolved in cyclohexane, and then 3 mmol of β-Alanine was added under the condition of an ice bath for 2 h. During the reaction, 4 M NaOH was used to adjust the pH of the solution to 9. The reaction solution was adjusted to pH 4 with 4 M HCl and then extracted with 30 mL ethyl acetate three times. After repeated washing with 0.1 M HCl and 1 M NaHCO_3_, the hapten product was obtained. The chemical structure of the product was characterized and verified by ^1^H NMR. The immunogen and coating antigen were prepared by coupling hapten with BSA and OVA using the carbodiimide method and were confirmed by UV-vis spectroscopy [27,28].

### 2.4. Preparation of MAb and Eu^3+^-Labeled MAb

The antiquinclorac MAb was prepared by classical hybridoma fusion and immune antibody preparation techniques [29]. Firstly, BALB/c mice (6 weeks old) were intraperitoneally injected with emulsified immunogen to screen spleen donors. Secondly, hybridoma cells were prepared by fusing spleen cells with SP2/0 myeloma cells and were screened by the limited dilution method. Finally, hybridoma cells with specificity and sensitivity were injected into the abdominal cavity of mice to prepare ascites and then purified into monoclonal antibodies by saturated ammonium sulfate precipitation. The Eu^3+^-labeled MAb was prepared according to our previously reported protocol and the similar literature [30,31].

### 2.5. Procedures of ELISA and TRFIA

For ELISA, the coated antigen diluted by CBS (100 μL/well) was added into 96-well plates and coated overnight at 4 °C. Then, 1% OVA blocking solution (300 μL/well) was added and blocked for 0.5 h. Next, quinclorac standard solution or sample extraction solution (50 μL/well) and PBS diluted MAb (50 μL/well) were added for competitive reaction for 1 h. PBS diluted HRP-GAR-IgG (100 μL/well) was added and incubated for 1 h. TMB solution (100 μL/well) was added and incubated for 20 min. Finally, 2 mol/L H_2_SO_4_ (50 μL/well) was added to terminate the reaction, and the absorbance at 450 nm was determined.

For TRFIA, the procedures of coating and blocking step were the same as ELISA, while the steps of immunoreactions by MAb and GAM-IgG in ELISA were replaced by the Eu^3+^-labeled MAb. Then, the enhancement solution (200 μL/well) was added and incubated for 8 min after gentle mixing, and the fluorescence intensity was detected.

Except for the special instructions, all incubation steps were carried out at 37 °C and washed four times with PBST after each step.

### 2.6. Optimization of ELISA and TRFIA

The optimal concentration of MAb and coating antigen was determined by the chessboard method. The effects of the amount of methanol or Na^+^ and pH values of PBS buffer solution were studied. The evaluation indexes were the half-maximal inhibition concentration (IC_50_) and maximum absorbance (A_max_) for ELISA, and IC_50_ and maximum fluorescence intensity (F_max_) for TRFIA. The optimal parameters were determined by considering the lowest IC_50_ and the highest A_max_/IC_50_ (F_max_/IC_50_). The standard curve was fitted by A/A_0_ (F/F_0_) against the logarithm of the quinclorac concentrations. The A and F were the absorbance and fluorescence signals present in quinclorac, while A_0_ and F_0_ were the absorbance and fluorescence signals without the presence of quinclorac. The sensitivity parameter (IC_50_, IC_20–80_) was calculated by the four-parameter logic equation (Y = A2 + (A1 − A2)/(1 + X/X0)^p^)) fitted by the Origin V8.0.

### 2.7. Cross-Reactivity of ELISA and TRFIA

The established ELISA and TRFIA were used to detect quinclorac analogs, and the IC_50_ values were calculated. The specificity of the method was evaluated by calculating the cross-reaction (CR). The CR is defined as: CR% = (IC_50_ of quinclorac/IC_50_ of analogs) × 100. A smaller CR value indicates better specificity of the method.

### 2.8. Analysis of Spiked Samples

Four types of environmental and agricultural samples (river water, paddy water, paddy soil, and brown rice) were collected in Sanming City, Fujian Province (China). It was verified by UPLC-MS/MS that quinclorac was nondetectable in the sample. The spiked concentrations of quinclorac were 1, 0.5, and 0.2 mg/kg for ELISA and 1, 0.2, and 0.05 mg/kg for TRFIA. After adding a series of quinclorac standards into the blank samples, the samples were mixed well and stayed overnight to simulate the interactions between quinclorac and the sample matrix. The river water and paddy water were filtered through a 0.22 um membrane and diluted twice with PBS prior to analysis. Then, 5 mL of 0.05% formic acid aqueous solution and 10 mL of methanol were added to 10 g of paddy soil (or brown rice) successively. The sample was vortex-treated (2000 rpm, 10 min), extracted by ultrasonic (100 Hz, 10 min), and centrifuged (5000 rpm, 5 min). After this, 1 mL of supernatant was filtered and diluted five times with PBS prior to determination. All samples were analyzed three times.

### 2.9. Real-Sample Detection and UPLC-MS/MS Validation

Twenty-four environmental and agricultural samples with a history of quinclorac application were collected from Sanming City, Fujian Province (China). The samples were extracted by the above methods and detected by ELISA, TRFIA, and UPLC-MS/MS.

## 3. Results and Discussion

### 3.1. Identification of Antigen and MAb

In the ^1^H-NMR spectrum (Figure S1, Supplementary Material), the synthesized compound showed the characteristic chemical shift of the target compound, indicating that quinclorac hapten was synthesized successfully. The conjugation between the hapten and BSA or OVA was confirmed by UV absorption spectra (Figures S2 and S3), showing characteristic peaks of hapten and BSA or OVA. The prepared MAb was identified by the SDS-PAGE, which showed a high purity and a molecular weight of about 150 kDa (Figure S4).

### 3.2. Optimal Parameters of ELISA and TRFIA

Both ELISA and TRFIA were based on the immune affinity reaction of antigen and antibody, and the factors in the reaction system had a great influence on the performance of the method [32]. Therefore, appropriate parameters might improve the performance of the detection method. Through comprehensive evaluation of IC_50_ and A_max_/IC_50_ (F_max_/IC_50_), the optimal parameters of ELISA were 0.25 mg/L coating antigen, 2.50 mg/L MAb, 5% methanol, 0.4 mol/L Na^+^, and pH 7.5, while the optimal parameters of TRFIA were 0.25 mg/L coating antigen, 2.50 mg/L Eu^3+^-labeled MAb, 10% methanol, 0.3 mol/L Na^+^, and pH 7.5 (Table S1).

### 3.3. Performance of ELISA and TRFIA

**Figure 2:**
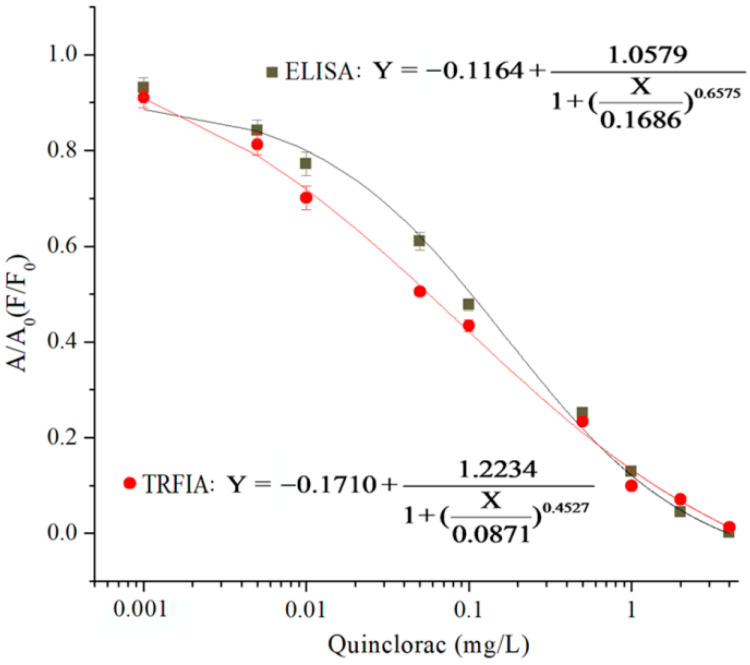
The standard curves of ELISA and TRFIA for quinclorac (*n* = 3).

With the optimized parameters, the standard curves of ELISA and TRFIA were established. There was a good correlation between the concentration of quinclorac and the signal intensity, and the sensitivity parameters were obtained by the four-parameter logic equation (Figure 2). For ELISA, the detection limit (LOD, IC_20_, 20 % inhibition concentration of quinclorac for the detection method) and IC_50_ were 0.020 mg/L and 0.169 mg/L, and the linear range (IC_20_–IC_80_, a range of 20 to 80 percent inhibition concentration of quinclorac for the detection method) was 0.020–1.389 mg /L. For TRFIA, the LOD and IC_50_ were 0.004 mg/L and 0.087 mg/L, and the linear range (IC_20_–IC_80_) was 0.004–1.861 mg/L.

Compared with ELISA, the IC_20_ and IC_50_ of TRFIA had improved 5-fold and 2-fold, respectively, showing a significantly improved sensitivity for the quinclorac detection. In addition, the linear range of TRFIA was broader than that of ELISA. The lanthanide chelates had the characteristics of a large Stokes shift, long decay time, wide excitation spectrum, and narrow emission spectrum. TRFIA achieved the specific detection of quinclorac, which benefited from the time-resolved fluorescence signal that effectively eliminated the interference of nonspecific fluorescence. Moreover, the enhanced dissociation can significantly improve the fluorescence signal and make TRFIA obtain a higher sensitivity and broader linear range [22]. The maximum residue limit (MRL) of quinclorac in brown rice was 1.0 mg/kg, so the sensitivity of ELISA and TRFIA could meet the requirements of the detection of quinclorac. In addition, TRFIA showed more obvious superiority than ELISA. ELISA required more than 2 h of immunoreaction time through at least five steps. TRFIA only needed 1 h of immunoreaction time by three steps, which could be efficiently and rapidly performed by the direct competition mode. In the procedure of TRFIA, the consumption of coating antigens and MAb was further reduced than in ELISA method. Therefore, the proposed TRFIA might be one of the potential detection methods with many advantages, such as higher sensitivity, broader linear range, simplicity, speediness, high efficiency, and low cost.

### 3.4. Specificity

The IC_50_ and CR of quinclorac analogs by ELISA and TRFIA are shown in Table 1. The results show that the CRs of quinclorac and its analogs were less than 2%. Therefore, MAb had a great specificity in recognizing quinclorac, and ELISA and TRFIA could be used to detect quinclorac without interference from its analogs.

### 3.5. Analysis of Spiked Environmental Samples

When detecting the environmental samples by ELISA and TRFIA, the matrix effects were one of the most common challenges, which might significantly reduce sensitivity and accuracy. To minimize the matrix effects from the environmental samples (including river water, paddy water, paddy soil, and brown rice), the dilution method was used as the easiest and most immediate strategy [33]. In this study, the water and rice water samples were diluted two times, and the soil and brown rice extracts were diluted five times. By this method, reliable results were obtained. The recoveries of ELISA were 77.7–101.4% with RSDs of 1.7–11.5%, while the recoveries of TRFIA were 77.3–106.1% with RSDs of 1.8–12.5% (Table 2). Therefore, the two methods could be applied to the accurate and reliable determination of quinclorac in environmental and agricultural samples.

### 3.6. Real-Sample Detection by ELISA, TRFIA and UPLC-MS/MS

**Figure 3:**
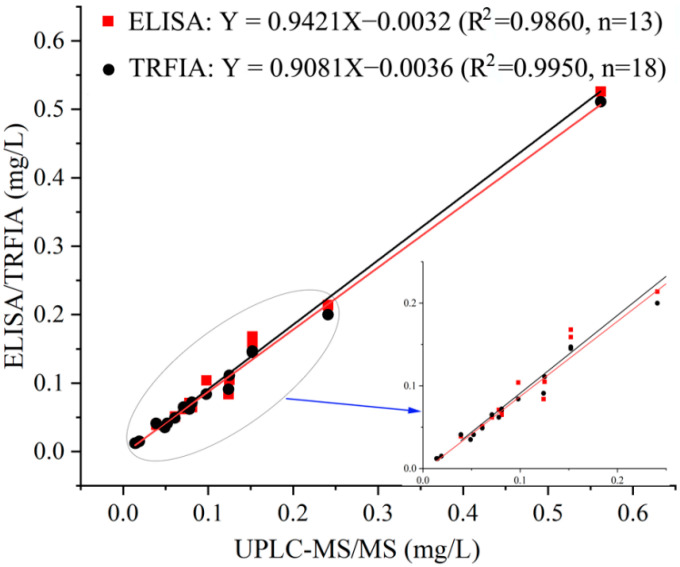
The correlation between ELISA, TRFIA, and UPLC-MS/MS for quinclorac.

To verify the practicability of the developed ELISA and TRFIA, the quinclorac residue in the real samples was detected by ELISA and TRFIA and referenced by UPLC-MS/MS. The quinclorac residue was detected in all samples except the river water samples. In addition, TRFIA and UPLC-MS/MS had lower LODs than that of ELISA for some samples that contained trace quinclorac. The LOD of UPLC-MS/MS was 5.0 μg/L. The results of ELISA, TRFIA, and UPLC-MS/MS showed good correlation, as the correlation coefficient (R^2^) of the linear regression equation was greater than 0.98 (Figure 3). These results further verify the reliability of the developed ELISA and TRFIA.

## 4. Conclusions

To further enhance detection ability and ensure food safety, ELISA and TRFIA based on MAb were successfully developed and applied for detecting quinclorac residue in environmental samples. The results of quinclorac residues could be rapid, and high throughput detected without using a complicated procedure and intensive labor. ELISA and TRFIA had good sensitivity, specificity, and reliability, which can meet the requirements of the detection of quinclorac. In addition, TRFIA showed better sensitivity and linear range than ELISA. The sensitivities of TRFIA were enhanced appreciably and benefited from using Eu^3+^-labeled Mab, which demonstrated its potential for improving the sensitivity and reducing interferences. Moreover, the developed TRFIA shortened the overall analytical procedure, testing time, and workload compared with ELISA and instrument-based detection methods. The established ELISA and TRFIA were used for the analysis of real environmental and agricultural samples, and the results showed great consistency with the referenced method of UPLC-MS/MS. Therefore, the established immunoassays could be considered alternative, efficient, rapid, and economical methods for the large-scale screening of quinclorac residues in environmental and agricultural products.

## Figures and Tables

**Table 1:** The cross-reactivity of analogs related to quinclorac by ELISA and TRFIA.

Compound	Structure	ELISA		TRFIA	
		IC_50_ (mg/L)	CR (%)	IC_50_ (mg/L)	CR (%)
Quinclorac	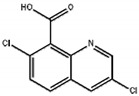	0.169	100	0.087	100
7-chloro-8-methylquinoline	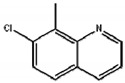	9.8	1.7	4.9	1.8
2-quinoline carboxylic acid	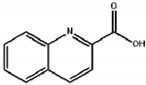	>50	<0.5	>50	<0.5
Quinmerac	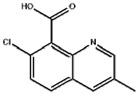	10.7	1.6	6.8	1.3

**Table 2:** The recoveries of quinclorac in spiked samples (*n* = 3).

Sample	ELISA			TRFIA		
	Concentration (mg/L or mg/kg)	Mean Recovery ± SD (%)	RSD (%)	Concentration (mg/L or mg/kg)	Mean Recovery ± SD (%)	RSD (%)
River water	1	88.7 ± 6.7	7.6	1	93.0 ± 9.2	9.9
	0.5	98.9 ± 5.6	5.7	0.2	103.3 ± 10.5	10.2
	0.2	88.0 ± 10.0	11.3	0.05	94.3 ± 8.5	9.0
Paddy water	1	101.4 ± 7.0	6.9	1	100.6 ± 12.2	12.1
	0.5	92.1 ± 6.0	6.5	0.2	106.1 ± 5.5	5.1
	0.2	87.6 ± 1.5	1.7	0.05	98.0 ± 1.8	1.8
Paddy soil	1	79.0 ± 3.9	5.0	1	86.0 ± 5.8	6.7
	0.5	83.2 ± 3.4	4.1	0.2	83.4 ± 10.4	12.5
	0.2	79.9 ± 9.2	11.5	0.05	77.3 ± 5.1	6.7
Brown rice	1	83.6 ± 6.2	7.5	1	82.7 ± 5.4	6.5
	0.5	87.5 ± 9.7	11.1	0.2	81.4 ± 7.4	9.1
	0.2	77.7 ± 6.2	8.0	0.05	79.0 ± 3.3	3.7

## Data Availability

The data presented in this study are available on request from the corresponding author.

## Supplementary Materials

The following supporting information can be downloaded at: www.mdpi.com/article/10.3390/bios12050338/s1, Figure S1: The ^1^H-NMR spectra of quinclorac hapten. Figure S2: The UV absorption spectra of hapten, coating antigen, and OVA. Figure S3: The UV absorption spectra of hapten, immunogen, and BSA. Figure S4: The SDS-PAGE of MAb. Table S1: The optimization of ELISA and TRFIA parameters for quinclorac.
