# Supplementary material for: Establishment and Comparative Analysis of Enzyme-Linked Immunoassay and Time-Resolved Fluoroimmunoassay for the Determination of Trace Quinclorac in Environment
Source: Biosensors (Basel). 2022 May 14;12(5):338. doi: 10.3390/bios12050338 (PMC9138993; doi:10.3390/bios12050338)
Supplement: Supplementary file 1 [file biosensors-12-00338-s001.zip › biosensors-1697533-supplementary.pdf]

Supplementary Material

Figure S1. The ^{1}H-NMR spectra of quinclorac hapten.

Figure S2. The UV absorption spectra of hapten, coating antigen and OVA.

Figure S3. The UV absorption spectra of hapten, immunogen and BSA.

Figure S4. The SDS-PAGE of MAb.

Table S1. The optimization of the ELISA and TRFIA parameters for quinclorac.

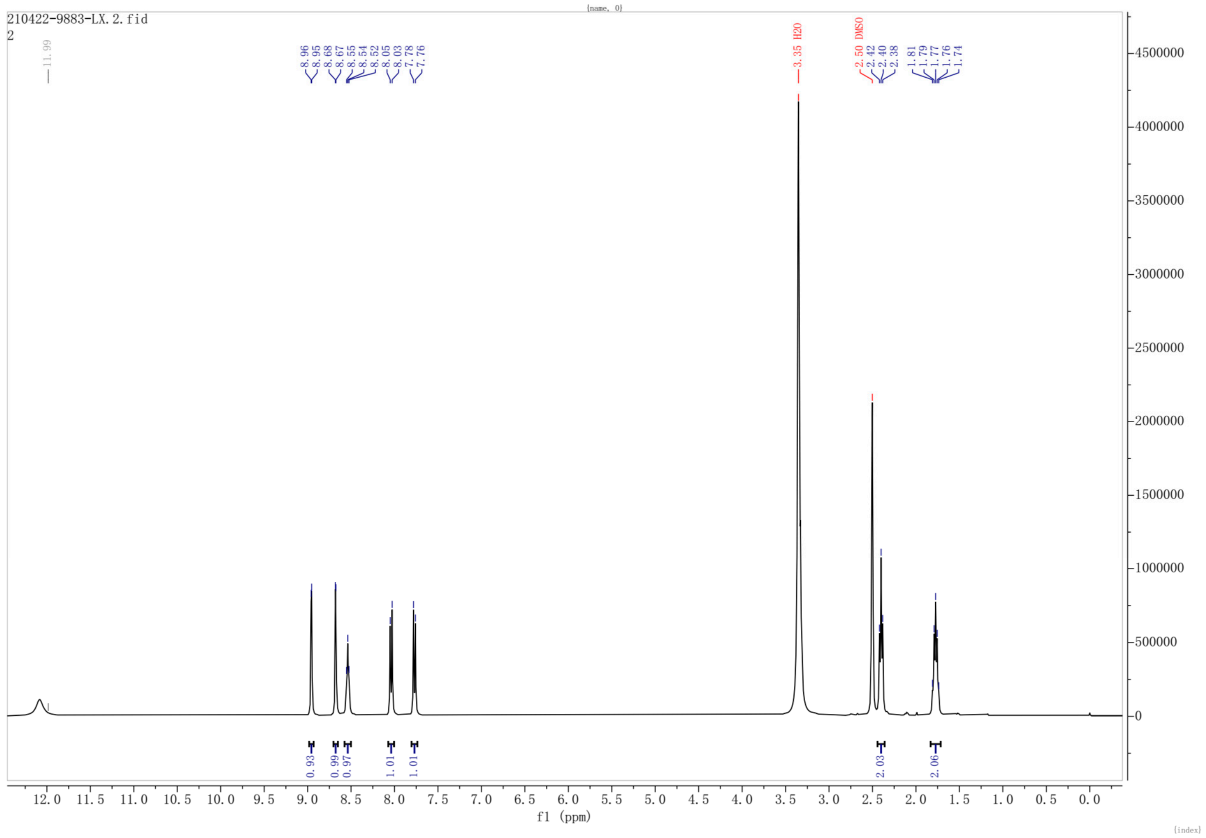

Figure S1. The ^{1}H-NMR spectra of quinclorac hapten.

^{1}H-NMR information: 1.81-1.74 ppm (2H，-CH_2)，2.42 - 2.38 ppm (2H，-CH_2)，7.78 - 7.76 ppm (1H, benzene ring), 8.05 - 8.03 ppm (1H, benzene ring), 8.55 - 8.52 ppm (1H，-NH-)，8.68 - 8.67 ppm (1H, pyridine ring), 8.95 - 8.96 ppm (1H, pyridine ring), 11.9 ppm (1H, -COOH).

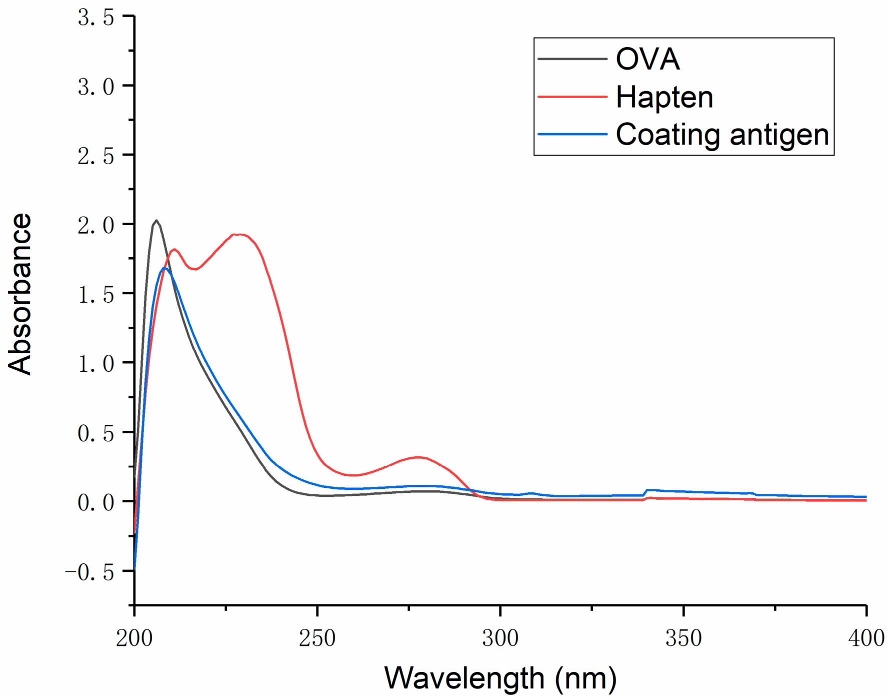

Figure S2. The UV absorption spectra of hapten, coating antigen and OVA.

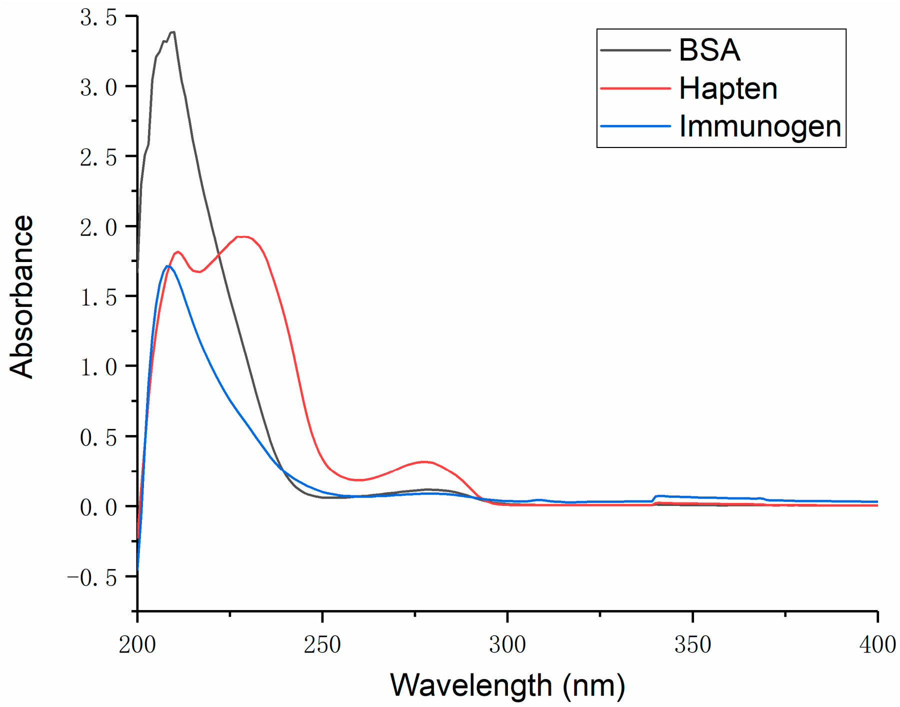

Figure S3. The UV absorption spectra of hapten, immunogen and BSA.

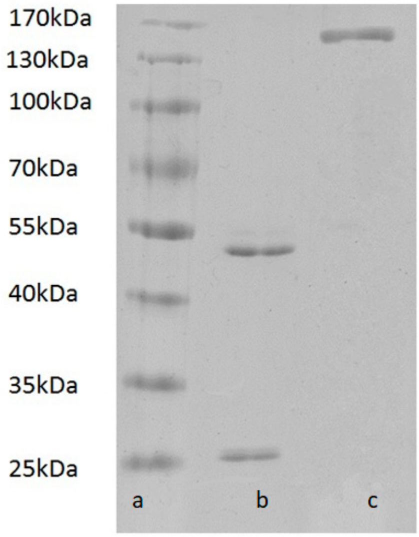

Figure S4. The SDS-PAGE of MAb.

(a: marker; b: denatured MAb; c: no denatured MAb)

Table S1. The optimization of the ELISA and TRFIA parameters for quinclorac.

Parameters		ELISA		TRFIA	
		IC_{50} (mg/L)	A_{max}/IC_{50}	IC_{50} (mg/L)	F_{max}/IC_{50}
Methanol (v/v,%)	0	0.453	5.32	1.152	17350
	5	0.164	14.6	0.135	128585
	10	0.172	13.9	0.101	248624
	20	0.210	10.7	0.323	41644
	30	0.370	7.05	0.755	16735
Na^{+} (mol/L)	0.1	0.271	8.79	0.251	82231
	0.2	0.205	10.4	0.172	100837
	0.3	0.179	12.5	0.124	130645
	0.4	0.159	14.1	0.111	109108
	0.5	0.243	9.28	0.285	47667
pH	5.5	0.456	2.55	0.286	88255
	6.5	0.446	4.96	0.289	72696
	7.5	0.179	12.5	0.088	223477
	8.5	0.215	9.04	0.104	193673
	9.5	0.450	5.03	1.458	17449
